# Phase Separation Regulates Metabolism, Mitochondria, and Diseases

**DOI:** 10.1002/mco2.70283

**Published:** 2025-07-01

**Authors:** Chuan Gao, Peng Ding, Changqing Zhang, Junjie Gao

**Affiliations:** ^1^ Department of Orthopaedics Shanghai Sixth People's Hospital Affiliated to Shanghai Jiao Tong University School of Medicine Shanghai China

**Keywords:** diseases, metabolism, mitochondria, phase separation

## Abstract

As a physicochemical mechanism, phase separation is a spatial and temporal regulator of specific molecules within a cell, and it provides a new perspective for understanding cellular pathophysiology. Phase separation is closely associated with multiple metabolic processes in the body, including the regulation of key metabolic enzymes and the physiology of mitochondria. Mitochondria also regulate multiple physiological functions through phase separation, including protecting healthy mitochondria and mRNAs in oocytes and regulating crosstalk between nuclear and mitochondrial. Importantly, abnormal phase separation in vivo is associated with the development of diseases, including cancer, neurodegenerative diseases, endocrine disorders, skeletal system diseases, and infectious diseases. This review summarizes the relationship between phase separation and metabolism under both physiological and pathological conditions, as well as the therapeutic potential of phase separation in the treatment of relevant diseases, aiming to explore the possibility of treating diseases by regulating phase separation.

## Graphical abstract

Mitochondrion‐related liquid–liquid phase separation. (1) Phase separation mediates the self‐assembly of mitochondrial nucleoids (mt‐nucleoids). (2) Phase segregation mediates the formation of mitochondrial RNA granules (MRGs). (3) Phase separation mediates the formation of mitochondrial degradosomes. (4) Phase segregation is involved in mitophagy.

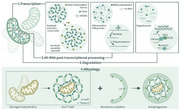

## 1 Introduction

Phase separation is a concept originally described in the field of materials chemistry, and it is widely believed that the molecular condensates formed by phase separation are temporal and spatial regulators of molecular distribution within a biological cell. Phase separation is considered the foundation of many biological processes, including the formation of some important cellular membraneless organelles (MLOs) [1], which are involved in cell signaling [2], nucleocytoplasmic transport [3], and cell development regulation [4, 5]. Phase separation regulates metabolism via several mechanisms. In anabolism and catabolism, phase separation regulates the synthesis and decomposition of substances by changing the activity of enzymes and thereby regulating gluconeogenesis, lipid metabolism, amino acid metabolism, and nucleotide metabolism [6]. In energy metabolism, phase separation is involved in several physiological processes in mitochondria, including the self‐assembly of mitochondrial nucleoids (mt‐nucleoids) [7, 8], autophagy of dysfunctional mitochondria (mitophagy) [9], and the formation of mitochondrial RNA granules (MRGs) [10].

Abnormal phase separation is associated with many diseases, especially protein deposition diseases, including neurodegenerative diseases [11] and type II diabetes [12]. In addition to protein deposition diseases, phase separation is involved in oncogenesis, Paget's disease of bone (PDB), and infectious diseases [13, 14]. In contrast to pathological deposition due to abnormal protein phase separation, the role of phase separation in oncogenesis and infectious diseases is more complex, including the lengthening of telomeres in cancer cells [15, 16], immune escape, and virus multiplication [17]. Disruption of normal condensates leads to the loss of effectiveness of certain small‐molecule drugs [18], which means that biomolecular condensates formed by phase separation provide a cellular microenvironment where drugs exert effects, abnormalities in phase separation may be associated with drug resistance. Therefore, targeting phase separation abnormalities may show therapeutic potential.

## 2 Phase Separation

### 2.1 Development of Cellular Phase Separation

**FIGURE 1:**
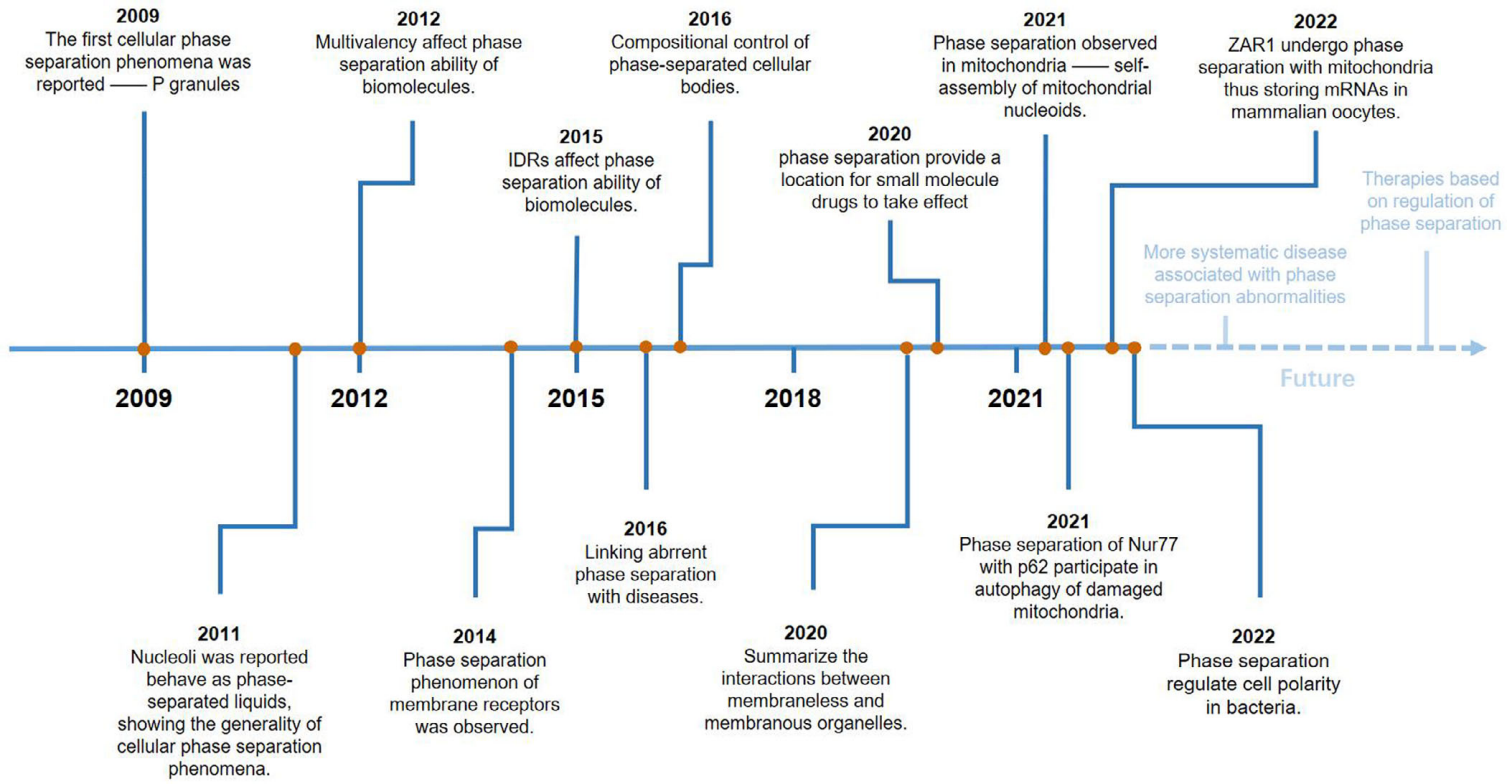
Development of cellular phase separation.

The concept of phase separation has been widely described in material chemistry. The first cellular phase separation events to be reported, in 2009, involved p granule formation [19]. Two years later, nucleoli were reported to behave as phase‐separated liquids, showing general cellular phase separation behavior [20]. Since then, an increasing number of phase separation behaviors have been observed within cells, but the influencing factors of intracellular phase separation are still unknown, and research on intracellular phase separation is limited to MLOs. In subsequent studies, intrinsically disordered regions (IDRs) and multivalency were shown to affect the phase separation ability of biomolecules [21, 22]. In addition, the phase separation of membrane receptors was observed in 2014, broadening the scope of phase separation research [23]. Since 2016, the relationship between phase separation and diseases has gradually attracted attention [24]. Diseases including neurodegenerative diseases, type II diabetes, PDB, infectious diseases, and cancer have been shown to be linked to aberrant phase separation in vivo [12, 13, 25]. While identifying the pathogenesis of diseases, further exploration into the relationship between phase separation and the treatment of diseases is most important. In 2020, research showed that phase separation provided a cellular microenvironment where small‐molecule antitumor drugs take effect, revealing a close link between biomolecular condensates formed by phase separation and disease treatment [18]. In regard to mitochondria, phase separation was first observed in the self‐assembly of mt‐nucleoids in 2021 [8]. More mitochondrion‐related phase separation has been reported in, for example, mitophagy and the formation of MRGs [9, 10]. In recent years, the focus of research has turned to phase separation and cell development. Studies in 2022 have proven that phase separation is involved in mRNAs storage in mammalian oocytes and cell polarity regulation in bacteria [4, 5] (Figure 1).

### 2.2 The Phase Separation Concept

**FIGURE 2:**
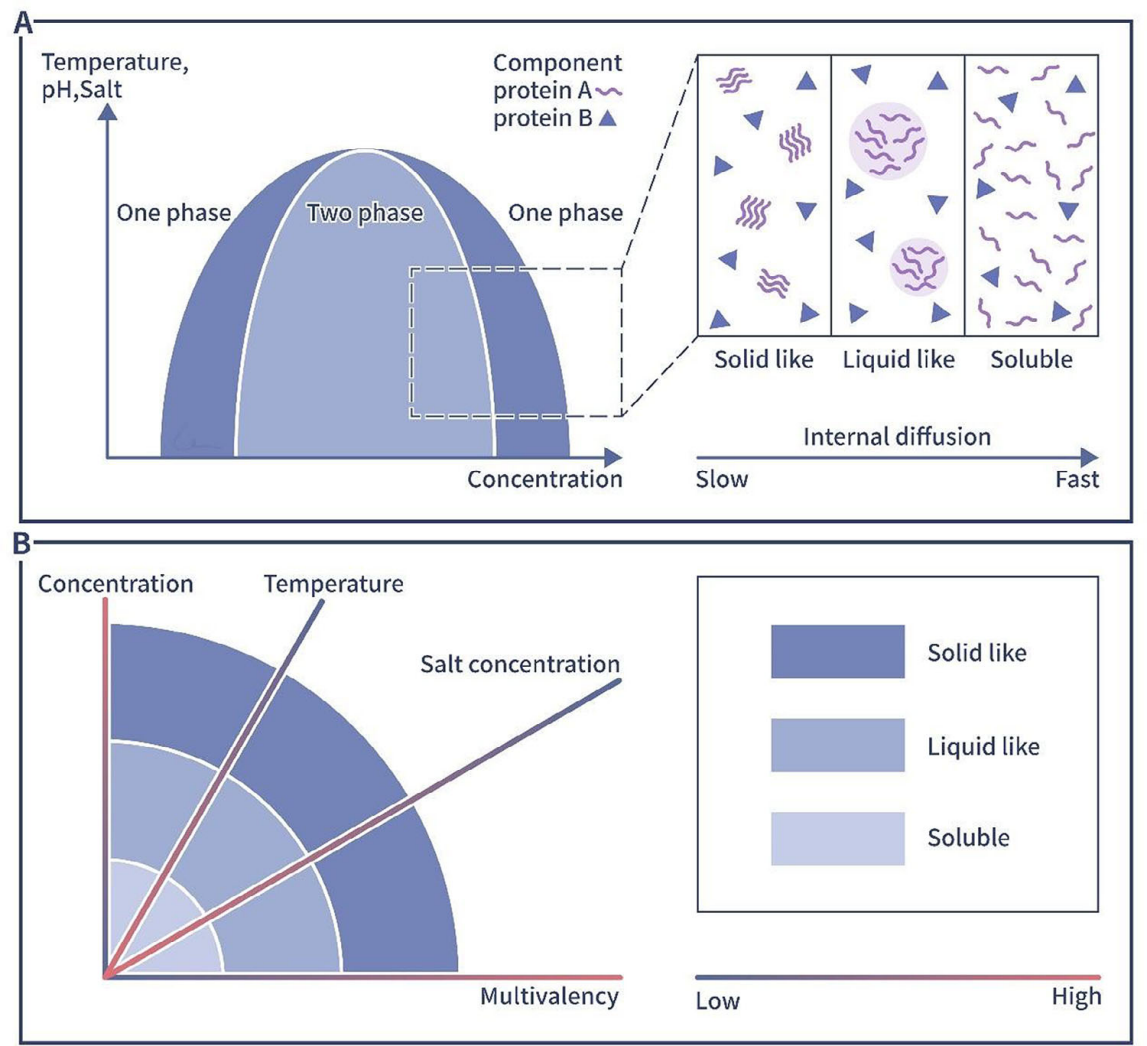
(A) Diagram showing phase separation in a two‐component system. When the concentration of component B is fixed and the temperature, pH, and salt ion concentration are suitable, the concentration of component A is changed, and the two components of the system change, from A dissolving in B (one phase) to liquid‐like condensate formation to A separating from B (two phases). During this process, the diffusion rate of the A molecule into the B component is continuously reduced. (B) Factors that affect protein phase separation: (1) multivalency: the higher the multivalency is, the faster solid‐like condensates form; (2) molecular concentration: the higher the concentration of molecules is, the faster solid‐like condensates form; (3) temperature: in the temperature range that does not lead to protein denaturation, a protein becomes more soluble as the temperature increases; and (4) salt concentration: before reaching the salting‐out concentration, a protein becomes more soluble as the salt ion concentration increases.

The concept of phase separation originally appeared in the material chemistry research and describes the transformation of an original stable state into a partially stable or unstable state in a multicomponent mixed system when external conditions such as temperature and pressure are changed, resulting in the separation of an originally compatible system mixture into two incompatible liquid phases or into a liquid and a solid phase. The former process refers to liquid–liquid phase separation (LLPS), and the latter process refers to solid–liquid phase separation. In recent years, with increasing interdisciplinarity in life sciences and materials chemistry, phase separation concepts have been widely applied to life sciences studies, in which it describes biological behaviors related to specific biomolecule aggregation to form biomolecule‐enriched condensates, with the concentration of the same biomolecular components outside the condensates being significantly lower [1, 26]. These biomolecular condensates constitute the dense phase, and the low‐density area outside a condensate is called the dilute phase [1, 13]. In summary, phase separation drives the inhomogeneous distribution of molecules in cells, playing specific biological roles in many physiological processes (Figure 2).

The first description of phase separation in cells was applied to p granule formation. Specifically, p granules show liquid‐like behaviors, including fusion, dripping, and wetting [19]. Since p granules have been described, an increasing number of phase separation behaviors have been observed within cells, including in the formation of heterochromatin [27, 28], part of MLOs such as stress granules (SGs) [29, 30], nucleoli [31], intercellular junctions such as tight junctions [32], and transmembrane channels such as nuclear pore complexes [3]. Different forms of phase separation, including liquid–liquid, liquid–gel, liquid–semicrystalline, or liquid–crystalline phase separation, have also been observed both in vitro and ex vivo. However, most cellular phase separation events involve liquid–liquid and liquid–gel phase separation [33].

Studies have shown that phase separation plays an important role in many biological processes. The formation of droplets through phase separation contributes to the spatial and temporal regulation of biological reactions, with these reactions relying on the formation of specific environments where enzymes and their potential substrates are concentrated. Phase separation also regulates the rate of biological reactions by isolating unwanted molecules [34]. Biomolecular condensates participate in protein transport, as ribonucleoprotein vesicles and nuclear pore complexes are formed by phase separation [3]. Phase separation also drives the formation of bridging granule junctions [32], which are essential for sealing tissues, cellular polarity, and intercellular signaling. Under stress conditions, phase separation forms SGs that participate in cellular responses to stress [29, 30]. In mitochondria, phase separation mediates the self‐assembly of mitochondrial nucleoids, thereby regulating mitochondrial DNA (mt‐DNA) transcription [8, 35]. Phase separation also regulates the development of cells by enabling mRNA and mitochondria storage in mammalian oocytes and cell polarity regulation in bacteria [4, 5, 36].

In addition to MLOs within the cytoplasm and nucleoplasm, condensates formed by phase separation are found on the cell membrane. Signaling domains enriched in receptors, adaptors, effectors, and specific lipids are observed on the plasma membrane [2]. Condensates formed on the cell membrane by certain proteins and lipids form larger areas of molecular concentration and greater fluid properties and are influenced by local curvature of the cell membrane. Thus, condensates on the cell membrane exhibit a lower condensate‐inducing protein concentration threshold and exhibit specific functions that are important for signal transduction mediated via signaling molecules in the membrane [2]. For example, T cell receptor on the plasma membrane can form phase separation‐mediated biomolecular condensates known as T cell microclusters. T cell microclusters include the transmembrane receptors T‐cell receptor (TCR), cluster of differentiation 28 antugen (CD28), and programmed cell death 1 (PD1); the kinases lymphocyte‐specific protein tyrosine kinase (LCK) and zeta‐chain associated protein kinase 70kDa (ZAP70); the adaptor proteins linker of activation of T cells (LAT), growth factor receptor‐bound protein‐2 (GRB2), lymphocyte cytosolic protein 2 (LCP2), and noncatalytic region of tyrosine kinase adaptor protein 1 (NCK1); and the enzymes son of sevenless homolog1 (SOS1), phospholipase C‐γ1 (PLCγ1), and cystathionine β‐Lyase (CBL). T cell microclusters usually have a higher density and lower mobility compared with the surrounding environment, thus increasing the likelihood of molecular interactions and facilitating the minimal binding time that is required for interactions to occur [2]. In addition to signaling at the cell membrane, phase separation phenomena are also observed in the regulation of intracellular molecular signaling. Specific molecules also aggregate in biomolecular condensates within cells. The formation of biomolecular condensates can increase the efficiency of signaling pathways. Phase separation widely participates in immune signaling such as innate immune receptors cyclic GMP–AMP synthase (cGAS)–stimulator of interferon genes (STING) and retinoic acid‐inducible gene‐I protein (RIG‐I) [2]. For example, in cGAS–STING signaling pathway, cGAS–dsDNA can form biomolecular condensates through phase separation and promote the production of cGAMP, which in turn activates STING, regulating the immune response [37]. In RIG‐I signaling pathway, tripartite motif‐containing protein 25 (TRIM25) can undergo phase separation thus recruiting RIG‐I into condensates and increasing its ubiquitylation by TRIM25. This process triggers the innate immune response against ssRNA viruses or dsRNA viruses [38]. Therefore, phase separation is involved in almost every step of cellular biological activities, and an in‐depth understanding of phase separation is important to the advancement of life sciences.

### 2.3 Characteristics of Phase‐Separated Biomolecules

Cellular phase separation is characterized by the characteristics of the biomolecules involved. The first criterion for LLPS is multivalency, which means, multiple binding sites are required [1]. The presence of multiple binding sites ensures that biomolecules are sufficiently concentrated to form liquid‐like droplets. The second criterion based on the three‐dimensional structure of a protein, which exerts a vital effect on LLPS. Intrinsically disordered proteins (IDPs), which contain IDRs, are thought to drive LLPS [39]. As IDPs cannot fold into a stable two‐ or three‐dimensional conformation under physiological conditions, they retain a certain degree of disorder after forming complexes and are more likely to form three‐dimensional molecular networks, which are necessary for the formation of biomolecular condensates. Experimental evidence has demonstrated that many biomolecular condensates contain IDPs [40]. Different biomolecular condensates are enriched in different IDRs [41]. However, not all IDPs can drive phase separation, and IDRs with a large electrical charge and/or enriched with proline residues show low biomolecular condensate formation efficacy [42, 43]. In contrast, although IDPs cannot form a stable two‐ or three‐dimensional conformation, their conformation plays an important role in IDR‐driven LLPS [44]. IDRs that tend to form spreading disordered linear structures exhibit greater capacity for LLPS than those that tend to form compact structures, which may be associated with reduced steric hindrance that exposes binding sites and promotes biomolecular condensate stability. IDRs usually carry specific repeat motifs with low sequence complexity that promote the formation of biomolecular condensates. For example, the disordered domain of nephrin is usually negatively charged and includes aromatic/hydrophobic residues [45]; Ddx4 carries a large number of phenylalanine–glycine and arginine–glycine (RG) dipeptide repeats in its N‐terminus [22], and the removal or mutation of these motifs may prevent LLPS [22, 45].

In addition, biomolecular condensates can be classified into scaffold and client molecules. Scaffold molecules form the bulk of biomolecular condensates, and client molecules are enriched in condensates by binding to free cognate sites in the scaffold [46]. The composition of biomolecular condensates can switch based on changes in scaffold concentration or valency [46]. Furthermore, aromatic residues can drive phase separation. Some specific aromatic residues drive LLPS in proteins lacking LLPS motifs, such as the intrinsically disordered C‐terminal domain of TAR DNA‐binding protein‐43 (TDP‐43), which lacks a dominant LLPS motif, TRP‐334 and two other tryptophan residues together mediate TDP‐43 LLPS [47]. TDP‐43 harbors a central α‐helical element that facilitates intermolecular self‐association and contributes to TDP‐43 LLPS [48].

### 2.4 Factors Regulating Phase Separation

Many factors, including temperature, salt levels, and biomolecule concentration, influence the phase separation capacity of molecules [49]. For example, as the concentration of protein increases, the protein will gradually form liquid like condensate. As the concentration increases further and higher, the liquid‐like condensate will form solid like aggregates [49].

Temperature is also an important factor that affects phase separation. Increasing the temperature initially promotes the occurrence of phase separation, but as the temperature increases further, the phase separation ability decreases, from low to high temperatures in a way similar to a parabolic curve. This is related to the thermodynamic properties of the molecules in biomolecular condensates [50]. Stress is a common factor that causes intracellular phase separation. Research shows that stress‐associated physiological conditions triggers poly(A)‐binding protein phase separation and forms biomolecular condensates in vitro which also called SGs. The formation of SGs is an adaptive response that can promote cell fitness during stress [29]. However, in living organisms, protein mutation might be the most common and primary reason for changes in protein condensate formation, as most intracellular studies focus on protein mutation [8, 51]. Differences in a few amino acids can lead to protein structure disorder, causing profound differences in cellular phase separation involving these proteins. This disorder is illustrated by studies on ApoE polymorphisms within a population [52]. The three isoforms of ApoE proteins, namely, ApoE2, ApoE3, and ApoE4, carry cysteine/arginine substitutions at positions 112 and 158, and thus, their phase separation efficacy differs. The ApoE2 isoform is more likely to form large condensates in cells and is also more likely to cause autophagosomal damage and complement‐mediated mitochondrial damage [52]. However, the ApoE4 isoform is resistant to phase separation, which means it forms fewer condensates and induces less damage than ApoE2. Some intracellularly produced small molecules can also have phase separation capacity. triphosphate. An example is triphosphate (ATP). ATP is a typical amphiphilic molecule that can prevent the formation of biomolecular condensates as well as dissolve previously formed protein aggregates [53, 54].

Inhibition of intracellular biomolecular condensate formation is important to study cellular LLPS. LLPS is driven by hydrophobicity and inhibited by electrostatic repulsion; therefore, researchers usually change the hydrophobicity between biomolecules to induce LLPS. Trans‐cyclohexane‐1,2‐diol, an aliphatic diol, was the first chemical reported to inhibit cellular LLPS [55] and was proven to inhibit the formation of the nuclear pore complex, thereby destroying the permeability barrier of the nuclear membrane; therefore, by using trans‐cyclohexane‐1,2‐diol, researchers can increase lipid‐mediated transfection of the nucleus in vitro [56]. In recent years, 1,6‐hexanediol (1,6‐HD), another aliphatic diol, has been widely used as an exogenous tool to inhibit intracellular biomolecular condensate formation in LLPS studies. Similar to trans‐cyclohexane‐1,2‐diol, 1,6‐HD interferes with the weak hydrophobic protein–protein/protein–RNA interactions required for liquid‐like droplet formation [57]. Thus, 1,6‐HD significantly inhibits cellular chromatin motion and hypercondensation. These effects are enhanced in a dose‐dependent manner [58]. In addition, 1,6‐HD can inhibit the LLPS of SARS‐CoV‐2 nucleocapsid (N) protein with viral RNA, thereby limiting the activation of the nuclear factor‐κB (NF‐κB) signaling pathway in host cells [59]. However, not all aliphatic diols show the ability to inhibit phase separation, and experimental evidence has indicated that the inhibitory effects of aliphatic diols are roughly proportional to their relative hydrophobicity [60].

Posttranslational modifications (PTMs) of proteins have an important effect on the ability of phase separation. PTMs mainly include phosphorylation, acetylation, poly(ADP‐ribosyl)‐ation (PAR), ubiquitination, and methylation. They can alter the ability of proteins to separate into biomolecular condensates by altering the covalent, noncovalent, electrostatic, hydrophobic, and hydrophilic forces of the proteins [61]. For example, PTMs can affect the phase separation ability of TDP‐43 and tau proteins, leading to neurodegenerative diseases [62].

### 2.5 Methods to Study Protein Phase Separation

It is generally believed that proteins with disordered sequences have the ability to form biomolecular condensates through phase separation. Since many sophisticated computational tools have been developed to analyze the structure and properties of proteins by inputting the sequence of the protein, we can predict the degree of disorder of proteins through protein sequences. PONDR score is often used to preanalyze the protein phase separation ability [8]; SEG is used to identify low‐complexity regions of the target protein [63]. LARKS is used to predict the potential of a sequence to form an amyloid‐like structure [64].

#### 2.5.1 Observe Phase Separation Intracellular and Extracellular Phenomenon

To study the phase separation ability of proteins, intracellular experiments and extracellular experiments are usually required. To localize biomolecular condensates in cells, a common method is to transfect plasmids with fluorophores so that cells express proteins with fluorophores and we can observe them under a confocal microscope. To observe whether a certain protein can form biomolecular condensates outside cells, the overexpression plasmid of this protein can be transfected into cells, and after extracting this protein, it is added to the extracellular system to observe its phase separation ability [8]. We can analyze the size and morphology of MLOs intracellular or extracellular under a confocal microscope. The diameters of MLOs within cells usually vary between a couple of hundred nanometers and a few micrometers. The size of the condensates determines the molecular exchange rate and other dynamic properties of the MLOs [65].

#### 2.5.2 Verify the Fluid Properties of Biomolecular Condensates

A photobleaching experiment is a method for intuitively observing the fluid properties of biomolecular condensates inside and outside cells. In short, after locating biomolecular aggregates inside and outside cells, part of the aggregates are bleached by strong light. If this aggregate has fluid properties, the bleached part will recover fluorescence intensity over time after being bleached as the unbleached protein molecules around flow to the bleached part [8]. The recovery of the bleached part reflects the molecular diffusion and exchange in these biomolecular condensates. The fusion of two or more biomolecular condensates is a strong indication of liquidity too. So pictures can also be taken under a confocal microscope to catch the moment when biomolecular condensates fuse [66].

#### 2.5.3 Identify the Components of Intracellular MLOs

It is hard but important to identify the components of intracellular MLOs as they are dynamic and the moleculars in biomolecular condensates change over time. Many methods have been developed to isolate the biomolecular condensates from the cell such as centrifugation, proximal labeling, sorting, and so on [67].

#### 2.5.4 Study Biological Functions of Phase‐Separated MLOs

To explore which domain of a protein determines the phase separation ability of the protein, different mutant proteins (truncation mutations or point mutations) carrying fluorophores are usually constructed according to different domains of the protein. After transfecting cells, we can observe whether the phase separation ability of the mutant protein changes. By transfecting cells with these mutant plasmids, we can observe the effects of proteins with different phase separation abilities on normal physiological functions or cellular pathology [51].

## 3 Metabolism and Phase Separation

### 3.1 Metabolism and Mitochondria

Metabolism is defined as a series of chemical reactions that lead to both the synthesis and degradation of biological molecules. Metabolism can be roughly classified into anabolism and catabolism. Catabolism produces energy by breaking down macromolecules into smaller molecules such as carbon dioxide, water, and ammonia, while anabolism consumes energy to synthesize required biomolecules such as nucleic acids, proteins, polysaccharides, and lipids that carry out cell functions [68].

#### 3.1.1 Enzymatic Regulation of Metabolism

Most cellular metabolism reactions are accelerated and regulated by enzymes [69]. Therefore, enzymatic activity directly affects metabolism. The mainstream view is that enzymatic activity is regulated through four mechanisms: allosteric regulation, covalent modification, zymogen activation, and regulatory protein regulation [70]. Through this process, the reaction rate of an enzyme and substrate can be directly changed to change the metabolic rate in organisms. In addition, cellular regulation of metabolism includes transcriptional and posttranscriptional processes, including PTMs, mediated by intracellular signaling pathways [71].

For a long time, various enzymes and substrates were believed to be evenly distributed in the matrix and to diffuse freely [69]. However, studies have shown that enzymes and substrates preferentially interact at specific times and locations [69]. The spatial and temporal differences in the distribution of enzymes form the premise of efficient metabolism. Biochemical condensates formed by phase separation can explain the distribution of enzymes, and an increasing number of phase separation events in various key enzymes in metabolic pathways have been identified [71]. This study provides a new direction for dynamically understanding intracellular metabolism.

#### 3.1.2 Mitochondrial Regulation of Metabolism

Mitochondria, organelles consisting of a bilayer membrane, are energy production sites where the tricarboxylic acid cycle occurs and where respiratory chains transfer electrons [71]. Electrons generated by catabolic pathways such as the tricarboxylic acid cycle, glycolysis, and fatty acid β‐oxidation enter the respiratory chain in the mitochondrial cristae to produce ATP in the form of NADH+H+ or FADH_2_. In cells with different functions, mitochondrial morphologies vary. In cardiomyocytes, which demand high energy levels to maintain functions, mitochondria are numerous and replete with cristae. In contrast, cells with low energy demands carry few mitochondria, and these mitochondria have a tubular morphology [72]. The tissue‐specific morphology of mitochondrial networks reflects the various on‐demand functions of these organelles [73]. In cells with different metabolic demands, mitochondria functions are regulated through mitochondrial fission and fusion and cell apoptosis [72, 74].

### 3.2 Phase Separation Regulates Cellular Metabolism

**FIGURE 3:**
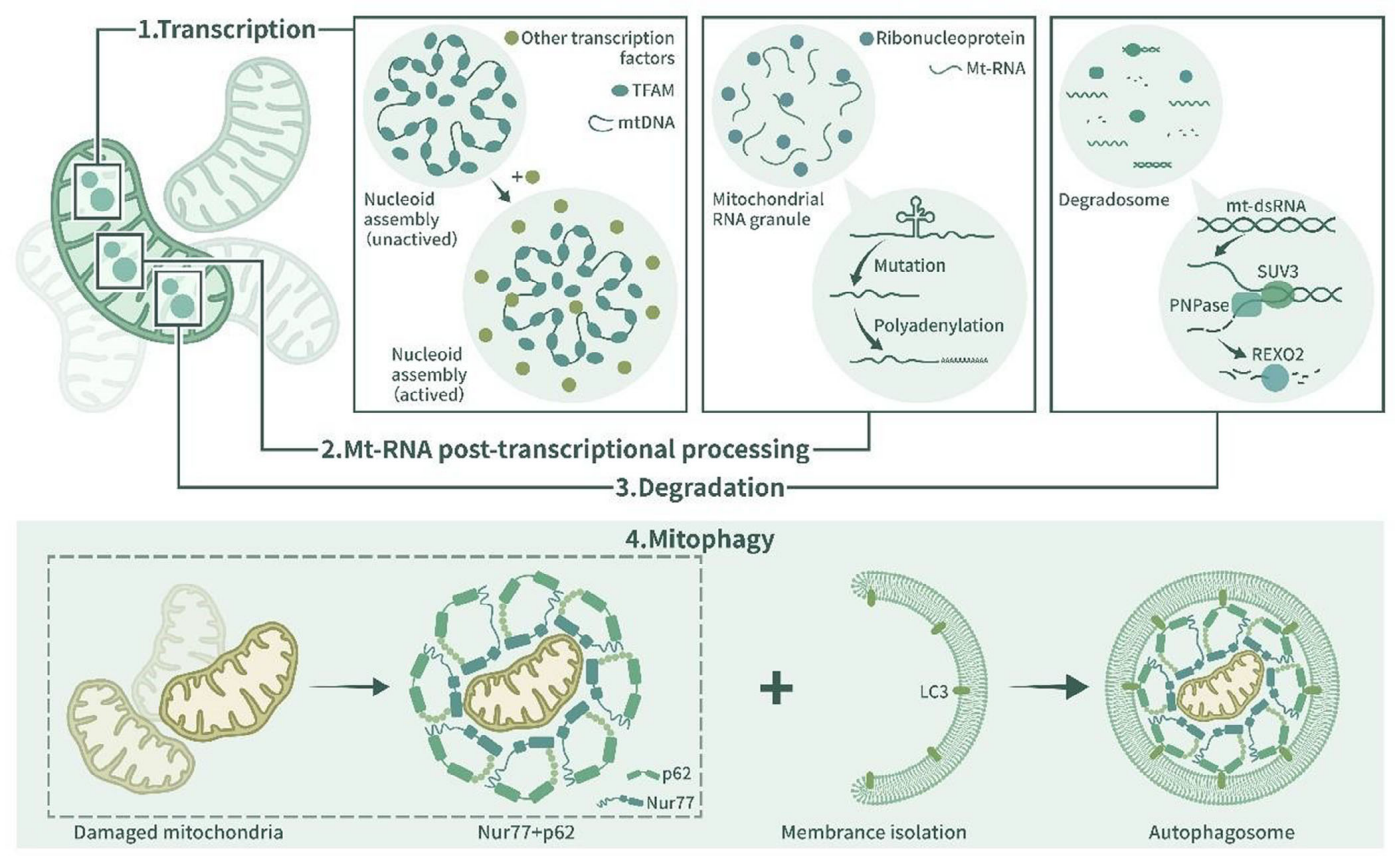
Mitochondrion‐related liquid–liquid phase separation. (1) Phase separation mediates the self‐assembly of mitochondrial nucleoids (mt‐nucleoids): mt‐nucleoids are formed by the phase separation of mitochondrial DNA (mt‐DNA) with TFAM. The components that make up the mt‐nucleoids change when nucleoid formation is in different stages. Mt‐nucleoids recruit factors including transcription initiation complexes, elongation complexes and termination factors through phase separation. (2) Phase segregation mediates the formation of mitochondrial RNA granules (MRGs): immature single‐stranded mRNA from mt‐nucleoids undergoes processing, maturation, polyadenylation and stabilization in MRGs. Mature mRNA transcripts are transferred from MRGs. (3) Phase separation mediates the formation of mitochondrial degradosomes: The degradosome is a site where mt‐dsRNA is degraded by SUV3, PNPase, and REXO2. SUV3 mediates double‐stranded RNA segregation, PNPase hydrolyses single‐stranded RNA into fragments, and REXO2 degrades RNA sequences comprising fewer than four nucleotides. (4) Phase segregation is involved in mitophagy: Nur77 undergoes phase separation with p62∖SQSTM1, thereby isolating damaged mitochondria and initiating mitophagy by binding LC3 on the autophagosome membrane.

When growth conditions change, diffuse enzymes often assemble and form discrete foci, which can be seen via light microscopy. Stimulus‐induced phase separation of many enzymes has been reported in many studies [75, 76, 77], suggesting that, in contrast to merely storing superfluous enzymes, biomolecular condensates may regulate enzymatic activity to control metabolism. Recent research shows that phase separation is involved in many mitochondrial physiological processes that may influence cellular metabolism [8, 35] (Figure 3).

#### 3.2.1 Phase Separation Regulates Metabolism Through Enzymatic Activity

When external energy is insufficient to maintain normal cellular physiological functions, cells enter a dormant quiescent state, also called the G0 phase, and activate catabolism‐ and stress tolerance‐related pathways [78]. During cellular dormancy, many cellular physicochemical properties, such as pH [79] and viscosity [80], are altered. Specifically, when energy levels are insufficient, cellular pH and volume decrease, leading to macromolecular crowding. Increased intracellular synthesis of trehalose and glycogen in dormant quiescent cells increases the viscosity of the cytoplasm. All of these factors reduce intracytoplasmic macromolecular diffusion, solidify the cytosol, and increase phase separation tendency, which drives many metabolic enzymes to assemble and form polymers spontaneously [80, 81].

Polymer formation mediated by phase separation regulates enzymatic activity and protects enzymes under stress conditions. In contrast to the irreversible aggregation of biochemical molecules, polymer formation is a dynamic process. When energy levels are sufficient, polymers disappear, and the cell re‐enters the cell cycle [82]. This phenomenon affects various metabolic pathways; for example, in yeast cells, condensation of the pyruvate kinase paralogue Cdc19 was observed during starvation. Cdc19 expression was associated with fermentation and found to be particularly important for glucose metabolism in yeast cells. Cdc19 carries a low complexity region (LCR). When several LCR residues are phosphorylated during glucose starvation or heat shock, Cdc19 undergoes phase separation and forms polymers [83]. The formation of the Cdc19 polymer protects Cdc19 from stress‐induced degradation, and when energy levels are restored, this polymer is solubilized, releasing Cdc19, which is vital to cell recovery [6].

Other phase separation events affecting enzymes related to glucose metabolism have been identified. For example, the glycolytic enzyme ATP‐dependent 6‐phospho‐fructokinase (PFK), a tetramer that is important in glycolysis because it catalyzes fructose‐6‐phosphate to generate fructose‐1,6‐bisphosphate, has been observed to assemble into biomolecular condensates in vitro, particularly in the presence of its substrate fructose 6‐phosphate [84, 85]. Glycolysis‐related enzymes, including ATP‐dependent‐6‐PFK‐1, enolase, fructose‐1,6‐bisphosphatase (FBPase), and alcohol dehydrogenases, form polymers under starvation conditions [6]. In addition, many glycolytic enzymes, including CDC19, are present in glycolytic bodies (G‐bodies). As typical biomolecular condensates, G‐bodies are generated when cells are in a hypoxic environment, and they enhance the local glycolysis rate to increase the local ATP concentration [86].

Phase separation of metabolism‐related enzymes has been reported in amino acid metabolism, lipid metabolism, and nucleotide metabolism. In amino acid metabolism, enzymes such as glutamine synthetase and cystathionine β‐synthase form filament polymers [87, 88]. Polymers of glutamine synthetase are induced by a decrease in intracellular pH during starvation, which inhibits glutamine activity. In lipid metabolism, acetyl‐CoA carboxylase (ACC), Fas1 and Fas2 subunits of fatty acid synthase (FAS), and many other enzymes have been shown to form condensates [89]. In nucleotide metabolism, human cytidine triphosphate (CTP) synthase, which is a key enzyme in the pyrimidine synthesis pathway, polymerizes into insoluble filaments, as observed by cryo‐electron microscopy, and the formation of polymers enhances the activity of CTP synthase [90]. Other enzymes observed to undergo phase separation within cells are listed in Table 1.

**TABLE 1:** Summary of enzymes associated with the four major metabolites observed to form biomolecular condensates in different species.

Enzyme	Metabolic functions	Substrate	Species	References
GDE	Glycogenolysis	Glycogen	Yeast	[6, 87]
6‐PFK‐1	Glycolysis	Fructose 6‐phosphate	Mammals/Yeast	[6, 87]
Pyruvate kinase	Glycolysis	Phosphoenolpyruvate	Mammals/Yeast	[6, 82]
Enolase	Glycolysis	2‐phosphoglycerate	Yeast	[6, 91]
FBPase	Glycolysis	Fructose‐1,6‐bisphosphate	Yeast	[6, 91]
Alcohol dehydrogenase	Alcohol metabolism	Alcohols	Yeast	[6, 82]
PEPCK1	Glycogen	Oxaloacetic acid	Mammals	[92, 93]
UTP‐glucose‐1‐phosphate uridylyltransferase	Lactose metabolism	Glucose‐1‐phosphate/UDP‐galactose	Yeast	[6, 82]
ACC	Fatty acids biosynthesis	Acetyl‐CoA	Mammals/Yeast	[6, 87]
Sterol‐3‐beta‐glucosyltransferase	Sterol metabolism	3‐beta‐hydroxysteroids	Yeast	[6, 82]
Asparagine synthetase	Amino acid synthesis	Aspartic acid	Yeast	[6, 87]
Glutamate synthetase	Amino acid synthesis	Alpha‐ketoglutaric acid/glutamine	Yeast	[6, 87]
Glutamate dehydrogenase	Energy metabolism	Glutamate	Mammals/yeast	[6, 87]
Glutamine synthetase	Amino acid synthesis/Nitrogen metabolism	Glutamate	Yeast/bacteria	[6, 87]
Glutaminase	Amino acid synthesis and Nitrogen metabolism	Glutamine	Drosophila	[6, 75]
CBS	Cystathionine synthesis	Serine/homocysteine	Yeast	[6, 82]
DAAO	Detoxification	D‐amino acid	Mammals	[92, 94]
CTP synthase	Pyrimidine synthesis	Uridine triphosphate	Mammals/drosophila/yeast/Bacteria	[90, 92]
AdSS	Pyrimidine synthesis	Inosinic acid/aspartic acid	Yeast	[6, 82]
IMPDH1, IMPDH2	Purine synthesis	Inosine monophosphate	Mammals	[92, 95]
PPAT enzyme	Purine synthesis	5‐phosphorlbosyl‐α‐pyrophosphate/glutamine	Mammals	[92, 96]
PFAS	Purine synthesis	Formylglycinamide ribonucleotide (FGAR)/glutamine	Mammals/yeast	[6, 82]
ADSL enzyme	Purine synthesis	Adenylosuccinate	Mammals	[6, 97]
RNR	Nucleotide metabolism	Ribonucleotides	Mammals	[92, 98]
XO	Purine catabolism and nitrogen metabolism	Hypoxanthine/xanthine	Mammals	[92, 99]
PRPP synthetase	Histidine, purine and pyrimidine biosynthesis	Ribose 5‐phosphate and ATP	Mammals	[88, 92]

Abbreviations: acetyl‐CoA carboxylase (ACC); adenylosuccinate lyase (ADSL); adenylosuccinate synthetase (AdSS); amidophosphoribosyltransferase (PPAT); ATP‐dependent‐6‐phosphofructokinase (6‐PFK‐1); cystathionine β‐synthase (CBS); cytidine triphosphate (CTP) synthase; D‐amino acid oxidase (DAAO); fructose‐1,6‐bisphosphatase (FBPase); glycogen debranching enzyme (GDE); inosine monophosphate dehydrogenase 1 and 2 (IMPDH1, IMPDH2); phosphoenolpyruvate carboxykinase 1 (PEPCK1); phosphoribosylformylglycinamidine synthase (PFAS); phosphoribosyl‐pyrophosphate (PRPP); ribonucleotide reductase (RNR); xanthine oxidase (XO).

#### 3.2.2 Phase Separation Regulates Metabolism Through Mitochondria

Phase separation has been observed in some physiological processes of mitochondria, including the self‐assembly of mt‐nucleoids [8, 35], mitophagy [9], and the formation of MRGs [10]. Mitochondrial nucleoids and MRGs are typical membrane‐free, liquid‐like biomolecular condensates [35, 100]. A mt‐nucleoid is an organizational unit of the mitochondrial genome. It contains mt‐DNA and architectural proteins that are vital to mitochondrial function. Mitochondrial transcription factor A (TFAM) undergoes phase separation with mt‐DNA, which explains the self‐assembly of mt‐nucleoids [8, 35]. Phase separation influences the size of mt‐nucleoids and is the primary physical mechanism in the self‐assembly of mt‐nucleoids. Under many kinds of cellular stress, mt‐nucleoids aggregate to form larger liquid‐like droplets [8], which is physiologically important, as the formation of nucleoid droplets promotes the recruitment of the transcription machinery, leading to the initiation of transcription, the enrichment of termination factors and the retention of substrates [35].

MRGs, which are located in the mitochondrial matrix, contain newly synthesized mRNA, double‐stranded RNA (dsRNA), RNA‐processing proteins and mitoribosome assembly factors [10, 101, 102]. MRGs are important to mitochondrial gene expression, as they participate in the mitochondrial posttranscriptional pathway. Relevant studies have shown that the formation of MRGs is related to mitochondrial RNA and proteins with disordered domains, and arbitrary deletion of both these RNAs and proteins leads to obstacles to MRG formation [103, 104]. The loss or aberrant accumulation of MRGs is often observed in dysfunctional mitochondria [102]. Increasing the number of mt‐nucleoids and MRGs through phase separation under cell stress conditions may enhance the electron transport function of the respiratory chain and thus increase energy output in response to stress.

Mitophagy is an evolutionarily conserved cellular process through which the quality and quantity of mitochondria are regulated [105]. It is vital to cellular energy metabolism, and it is generally believed that dysfunctional or superfluous mitochondria are removed from cells through the actions of the PINK1/Parkin‐mediated pathway or Parkin‐independent pathway [106]. Recent research has shown that in the presence of celastrol, the ubiquitinated mitochondrial nuclear receiver Nur77 (also called TR3, NGFI‐B, or NR4A1) undergoes phase separation with p62/sqstm1 and forms biochemical condensates that can sequester dysfunctional mitochondria and connect targeted mitochondria bound for mitophagy [9]. The function of Nur77 condensates is mediated by two domains, the C‐terminal ligand‐binding domain (LBD) and N‐terminal IDR of Nur77. Celastrol ubiquitinates the LBD, which mediates Nur77 and p62 biochemical condensate formation, which sequesters damaged mitochondria. In addition, the interaction between IDR and Phor and the Bem1p (PB1) domain in p62 enables condensates to connect with the outer membrane of autophagosomes and mediate autophagy [9]. This celastrol‐induced, p62‐mediated, and Parkin‐independent mitophagy pathway shows therapeutic potential for targeting phase separation‐regulated mitophagy.

Degradosomes consist of nucleases and other RNA decay‐associated proteins. In bacteria, ribonuclease (RNase) E forms the principal scaffold of the RNA degradosome and regulates mRNA decay [107, 108]. The disruption of degradosome formation can impede the growth of bacteria [109]. Studies have confirmed that RNase E degradosomes in bacterial cytoplasts and mRNA undergo phase separation from bacterial ribonucleoprotein bodies (BR bodies), and BR bodies form localized sites for RNA degradation [110]. The rate of bacterial mRNA decay is limited by RNase E, which forms a scaffold for BR bodies. Organization of BR bodies facilitates the rapid mRNA decay process in bacteria by organizing the RNA decay machinery together with mRNA substrates [107]. Mitochondrial degradosomes are sites of mt‐RNA degradation [108]. Their functions are related to proteins including SUV3, PNPase, and REXO2 [10]. The loss of SUV3, PNPase, or REXO2 leads to the accumulation of mt‐dsRNA foci in the matrix, disrupting mitochondrial function [10]. Whether degradosomes in mitochondria can form biochemical condensates with mt‐mRNA remains unknown; however, mitochondrial RNA degradation regulated by phase separation through degradosomes may be a potential mechanism through which to regulate mitochondrial energy metabolism.

## 4 Mitochondria Perform a Variety of Physiological Functions Through Phase Separation

Recently, research has shown that mitochondria undergo phase separation with RNA‐binding proteins (RBPs), including ZAR1, YBX2, DDX6, LSM14B, and 4E‐T (EIF4ENIF1), forming a hydrogel‐like matrix that provides a “shelter” for storing maternal mRNAs [4], which is vital for the survival of mammalian oocytes, as transcription stops during the final stages of oocyte growth, and new proteins can be translated only with these stored mRNAs [111, 112]. Research shows that the formation of a hydrogel‐like matrix depends on an increase in the mitochondrial membrane potential during oocyte growth. Blocking an increase in mitochondrial membrane potential with drugs such as antimycin A, FCCP, or oligomycin inhibited the formation of a hydrogel‐like matrix [4]. Likely, Balbiani body (Bb), a kind of MLO comprised of mitochondria and RNA, is widely observed in oocytes. Bb is considered to protect healthy mitochondria that will be passed on to the next generation [113]. The study of mitochondria, membrane‐bound organelles that undergo phase separation with proteins, has broadened our understanding of the function of mitochondria, and mitochondria are more than the sites of energy production.

The phase separation of Nur77 not only participates in mitochondrial autophagy, but also in mitochondria‐related apoptosis of tumor cells. XS561, a chemical compound, is a Nur77 ligand which can induce the translocation of Nur77 from the nucleus to mitochondria and form Nur77/Bcl‐2 condensates through phase separation, leading to mitochondria‐related apoptosis [114].

Phase separation also acts as a bridge between nuclear and mitochondrial communication and regulation [36]. Under normal conditions, the alternative reading frame (ARF) and nucleophosmin (NPM) in nucleolus form liquid condensates through phase separation, enriching ARF in nucleolus. Under stress conditions, mitochondrial cytochrome *c* (Cc) migrates to nucleus and competes with ARF for NPM binding. As a result, ARF, a tumor suppressor protein, is released from the nucleolus, performing its functions against DNA damage [115]. In addition, DNA damage also triggers nucleus‐translocated Cc to interacts with many histone chaperones in nucleolus, performing DNA repair through regulating nucleosome assembly activity and likely nuclear condensate formation [116]. All signs indicate that phase separation may play an important role in communication between different organelles.

## 5 Phase Separation and Diseases

**FIGURE 4:**
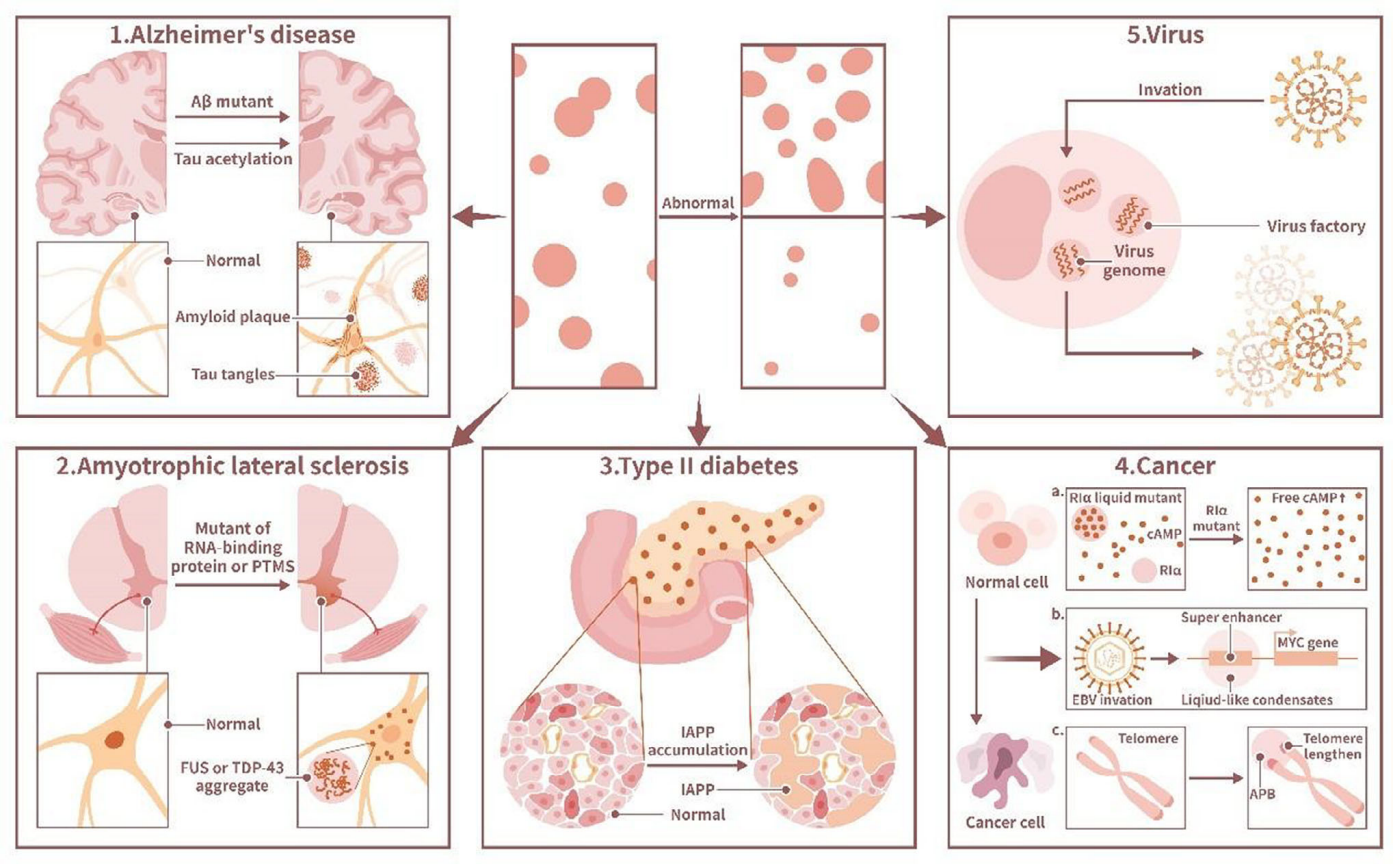
Phase separation and disease: Abnormal phase separation is observed in the pathology of many diseases. (1) Alzheimer's disease (AD): AD patients often present with mutations in Aβ proteins or with abnormal acetylation of TUA proteins, resulting in amyloid deposits and tau tangles. (2) Amyotrophic lateral sclerosis (ALS): Abnormal mutations in RNA‐binding proteins (RBPs, including the FUS protein, TDP‐43 proteins HNRNPA1, HNRNPA2, and TIA1) or abnormal posttranslational modifications (PTMs) lead to deposition of RBPs in spinal forefoot neurons. (3) Type II diabetes: Abnormal accumulation of misfolded islet amyloid polypeptides (IAPPs) leading to impaired islet function is a feature of type II diabetes. (4) Cancer: Phase separation promotes oncogenesis in several ways: (a) The concentration of the second messenger cAMP is regulated. Under normal conditions, cAMP is separated from the type I regulatory subunit RIa of the cAMP‐dependent protein kinase (PKA) and forms droplets in cells, and the concentration of free cAMP decreases. When RIa is mutated, droplet formation is inhibited, and the level of intracellular free cAMP increases, causing oncogenesis. (b) Tumor‐associated viruses, such as EBV, invades host cells and forms liquid‐like condensates on the superenhancers in the MYC gene, leading to oncogenesis. (c) Acute promyelocytic leukemia (APL) cells rely on phase separation to form ALT‐associated promyelocytic leukemia nucleosomes (APBs) to extend telomeres. (5) Viruses: After invading host cells, a virus forms viral DNA factories through phase separation in the cytoplasm, and these compartments contain the viral genome and promote viral replication.

The relationship between phase separation and diseases has gradually attracted attention in recent years as the discovery of cellular phase separation has broadened our mechanistic understanding of certain diseases. Diseases including oncogenesis, neurodegenerative diseases, endocrine disorders, skeletal system diseases, and infectious diseases have been shown to be linked to aberrant phase separation in vivo. Therefore, it is vital to expound how aberrant phase separation causes these diseases (Figure 4).

### 5.1 The Central Nervous System

Neurodegenerative diseases, in which patients present with chronic progressive cognitive or motor deterioration, include mainly Alzheimer's disease (AD), amyotrophic lateral sclerosis (ALS), frontotemporal dementia (FTD), Parkinson's disease (PD), and Huntington's disease (HD). They are characterized by progressive loss of neurons with organic and functional changes in the brain and peripheral organs [117]. The neural damage of most neurodegenerative diseases is caused mainly by the abnormal accumulation of misfolded cytosolic or nuclear proteins, which thus function as pathological markers [118]. Examples include the aggregation of tau protein or amyloid‐β (Aβ) in AD, aggregation of RBPs TDP‐43 or fused in sarcoma (FUS) in ALS and FTD, α‐Synuclein (α‐Syn) aggregation and amyloid formation in PD and polyglutamine (polyQ) aggregation in HD [13, 119]. Aberrant protein aggregation may spread among neurons along anatomically connected pathways, which corresponds to the progression of neurodegenerative diseases [120]. Most of the aforementioned proteins above undergo spontaneous phase separation when coding genes undergo mutation or aberrant PTMs [13].

#### 5.1.1 Alzheimer's Disease

AD is a typical neurodegenerative disease associated with abnormal phase separation of tau proteins. Extracellular plaques composed of Aβ and intracellular neurofibrillary tangles (NFTs) formed by abnormally phosphorylated tau proteins are important pathological markers of AD [121]. The tau protein, which contains an IDR [122], is associated with the polymerization of tubulin, thus nucleating microtubule bundles and stabilizing microtubule structures [123]. Under pathological conditions, tau is phosphorylated and then dissociates from microtubules [124]. In vitro, the tau protein forms liquid‐like drops in which tubulin is enriched. Microtubule bundles are polymerized within these drops, causing tau drop deformation into long strip structures [125], suggesting that the phase separation of tau protein may play an important role in the microtubule‐associated function of tau.

PTMs regulate the phase separation of the tau protein in vivo. Phosphorylation promotes tau protein LLPS [62, 126], while acetylation inhibits tau protein LLPS in vitro [127]. Increased phosphorylation levels of the tau protein increase its aggregation rate [62]. Moreover, high‐molecular‐weight soluble and phosphorylated tau protein isolated from the human brain in AD has been shown to undergo phase separation and form aggregates [62]. Interactions between phosphorylated tau proteins and RNA; RBPs, including TIA‐1; and nucleoporins, such as Nup98, may drive this phase separation [128]. Moreover, the aberrant phase separation of phosphorylated tau protein in NFTs in vivo is thought to contribute to AD development. Specific biochemical molecules have also been associated with the occurrence of tau protein‐containing NFTs formed by aberrant phase separation, such as the calcium‐binding protein EFhd2 and chaperone protein disulfide isomerase [129, 130].

Aβ, a typical IDP [131], is another significant pathological marker in AD. Aβ may undergo heterogeneous nucleation with extrinsic factors within the brain parenchyma, which leads to the abnormal phase separation of Aβ and neuritic plaque formation [132]. The neuronal cell membrane is a major site of the neuronal toxicity induced Aβ peptides in AD, as lipid surfaces in the neuronal cell membrane are enriched with gangliosides and cholesterol, which are common heterogeneous nucleator agents for Aβ that undergoes abnormal phase separation [115]. Mutations in Aβ are associated with familial AD, and different Aβ mutations result in different phase separation capacities. PTMs of Aβ also affect the phase separation ability of Aβ and thus influence Aβ aggregate formation into polymers. For example, both Aβ, N3pE and pSer8Aβ exhibit enhanced aggregation at oligomers and form fibril aggregates. Both Aβ mutations and PTMs may be associated with the maturation of Aβ amyloid plaques in vivo [133]. During abnormal phase separation of Aβ, which leads to Aβ aggregate formation, the protein conformation of Aβ is altered. In vitro, during dimerization, Aβ42 undergoes random coil‐to‐β‐sheet transitions, which is considered a mechanism of Aβ aggregation [134]. Interestingly, the ganglioside GM1 exerts a neuroprotective effect by preventing abnormal phase separation by reducing the formation of Aβ42 dimers because it attenuates the tendency of Aβ42 to form β‐sheet structures [134]. Moreover, the toxicity induced by Aβ may be mediated by the heterogeneous nucleation of Aβ at neuronal membranes, clearly leading to the abnormal phase separation of Aβ and forming extracellular plaques from its original phase [132].

#### 5.1.2 Amyotrophic Lateral Sclerosis and Frontotemporal Dementia

Aberrant phase separation is involved in the pathological process of ALS and FTD. The aggregation of RBPs, especially FUS and TDP‐43, in the neuronal cytoplasm of patients is a pathological marker of ALS and FTD. Normally, FUS and TDP‐43 are enriched in the nucleus. SGs, which are RNA granules, are particularly important to the formation of ALS and FTD [135]. RBPs undergo LLPS with RNA to form RNA granules. Mutations in RBPs, including FUS, TDP‐43, HNRNPA1, HNRNPA2, and TIA1, lead to the accumulation of these proteins in SGs, which promotes the development of ALS [136, 137]. Moreover, several SG proteins, including PABPC1, TIA1, and EIF4G1, are observed in FUS and TDP‐43 aggregates in the brains of ALS and FTD patients. Some ALS‐linked mutations of RBPs change the characteristics of SGs [136, 138]. All of these findings imply that abnormal phase separation of RPBs in SGs, especially FUS and TDP‐43, may lead to ALS and FTD. The distinct prion‐like domains of FUS and TDP‐43 drive the formation of condensates [139]. The accumulation of FUS and TDP‐43 is caused by the disorder of their nuclear import, which is regulated by PTMs and the nuclear import receptor. K84 acetylation reduces TDP‐43 nuclear import, while K136 acetylation reduces its RNA binding and splicing capabilities. Thus, acetylation induces phase separation mediated via the C‐terminal low complexity domain in RBPs, which form insoluble aggregates with pathologically phosphorylated and ubiquitinated TDP‐43 [138, 140]. The mutation of the nuclear localization signal and loss of normal posttranslational arginine methylation disrupts the nuclear import of FUS by weakening the FUS–transportin interaction and promotes FUS LLPS driven by three arginine–glycine–glycine (RGG) repeat domains in RGG/RG motifs, leading to FUS aggregation [141, 142, 143]. Recent research has shown that sirtuin‐1 reduces TDP‐43 aggregation by deacetylating K136‐acetylated TDP‐43 [140], suggesting that regulating the PTMs of targeted proteins may be used to treat diseases linked to aberrant phase separation. Tau protein also participates in the formation of FTD. When the tau‐encoding MAPT gene on chromosome 17 is mutated, it results in FTD with parkinsonism (FTDP‐17) as the tau protein LLPS is promoted [128].

#### 5.1.3 Other Neurodegenerative Diseases

α‐Syn, a neuronal protein, is highly localized in presynaptic nerve terminals. Accumulation of misfolded a‐Syn is linked to neurodegenerative diseases, including PD, Lewy body dementia, multiple system atrophy, neurodegeneration with brain iron accumulation type I, diffuse Lewy body disease, and Lewy body variant of AD [144]. α‐Syn undergoes LLPS and a subsequent liquid‐to‐solid phase transition, which leads to amyloid fibril formation in vitro [145]. Factors including low pH, phosphomimetic substitution, and familial PD mutations promote α‐Syn LLPS and the aggregation of mature fibrils [145]. Recent studies have reported the formation of α‐Syn liquid‐like droplets in cells [119], which may explain the role phase separation plays in α‐Syn‐linked neurodegenerative diseases.

HD is caused by the expansion mutation of CAG and CTG sequences in the huntingtin (HTT) gene, which produces aberrant HTT polyQ tract expansion protein and additional expansion proteins, including poly‐Ala, poly‐Ser, poly‐Leu, and poly‐Cys [146, 147]. Aberrant HTT polyQ tract expansion proteins undergo phase separation, forming liquid‐like assemblies and then transforming into solid‐like fibrous assemblies. This phase transition is driven by the polyQ tract and proline‐rich region in these proteins, which form fibrous polymers that lead to neurotoxicity [148].

### 5.2 Endocrine Diseases

Relationships between LLPS and endocrine cells have been observed in recent years, and the potential role of this relationship in male sexual development has been discovered [149]. Aberrant LLPS in endocrine cells causes endocrinological diseases, such as type II diabetes. The accumulation of misfolded islet amyloid polypeptide (IAPP) in pancreatic β‐cells, which reduces its normal secretion function, is a hallmark of type II diabetes. Research has shown that IAPP undergoes LLPS, forming protein aggregates in β‐cells [12]. Insulin can delay the LLPS of IAPP [12]. Interestingly, type II diabetes caused by aberrant LLPS of IAPP may be connected to PD. Notably, IAPP has been shown to induce the formation of α‐Syn aggregates in vitro, which may explain why patients with type II diabetes are more likely to develop PD [150].

### 5.3 The Skeletal System

PDB, a chronic and focal bone disorder, is characterized by a series of symptoms, including bone pain, noticeable deformities and arthritis at adjacent joints, and fractures [25]. PDB is a genetically heterogeneous disorder, and approximately 25–50% of familial PDB patients present with a mutation in the SQSTM1 gene, which encodes p62 [151, 152]. Research has shown that p62 forms liquid‐like P62 bodies through phase separation in cells [151]. The p62 mutations in PDB disrupt p62 phase separation, which affects the formation of the p62 body as well as the autophagic degradation of p62. Abnormal p62 phase separation is currently regarded as the cause of PDB [151].

### 5.4 Infectious Diseases and Cellular Immune Responses

Phase separation events can be found throughout multiple stages of infection, including the invasion of host cells by pathogens and the host cell immune responses to pathogens. It involves the production of virus‐specific condensates [153, 154], alteration of MLOs in host cells [155, 156], and the production of membrane‐associated condensates (clusters) in immune signaling pathways formed by cell‐surface immune receptors and their ligands [2].

#### 5.4.1 Virus‐Specific Condensates

Relevant research has shown that viruses often form novel phase separation‐induced condensates that contain factors for viral genome replication and gene expression as well as components of an antiviral immune response in host cells. For example, the compartments formed upon infection by vesicular stomatitis virus (VSV) exhibit liquid‐like properties, and expression of three proteins in the VSV replication machinery in host cells is sufficient to drive the formation of a viral compartments via phase separation [153]. Similarly, inclusions called Negri bodies that form in the cytoplasm of rabies virus‐infected cells are liquid‐like organelles, and their formation is driven by an IDR in the rabies virus P protein, promoting viral genome replication [154].

#### 5.4.2 The Influence of MLOs in Host Cells

Many viruses affect the phase separation of intrinsic MLOs in host cells. Many viruses undergo LLPS and form condensates with RNA in the host cell cytoplasm and regulate the formation of membrane‐free organelles in host cells, including SGs and processing bodies (PBs). SGs store cellular mRNA, and PBs facilitate mRNA decay and processing, which together regulate mRNA expression. Viruses inhibit the cellular stress response, promoting viral replication by inhibiting the formation of SGs or PBs or by altering their composition. For example, viruses often cleave G3BP, a key protein required for the formation of SGs, and Dcap1, a key protein for PBs, interfering with normal phase separation [155, 156].

#### 5.4.3 Regulation of the Antiviral Responses of Immune Cells

Phase separation also plays an important role in the antiviral responses of immune cells. Immune cells maintain body homeostasis after pathogen invasion by breaking down pathogens in the cytoplasm, thereby terminating pathogenic stimuli. This process involves the activation of multiple immune signaling pathways, including the TCR and B‐cell receptor pathways, and cytosolic signaling pathways, including the cGAS–STING, RIG‐I, and NF‐κB pathways [2]. Disruption of the normal phase separation of these signaling pathway components may lead to immune response failure.

#### 5.4.4 COVID‐19

In recent years, COVID‐19 research has become a hot spot. SARS‐CoV‐2, similar to other coronaviruses, binds to the human receptor ACE2 to mediate virus entry into host cells [157]. Studies on COVID‐19 invasion driven by phase separation and damage to host cells have been reported. Among the various proteins that comprise SARS‐CoV‐2, the nucleocapsid (N) protein has been shown to be involved in the phase separation of multiple substances mediating multiple pathological processes [158, 159, 160]. Similar to other proteins involved in phase separation, the N protein is enriched with IDRs. The N‐protein consists of five structural domains, including the N‐terminal disordered region; the folded N‐terminal structural domain, which is involved in RNA binding; the junction domain followed by 73 amino acids; the dimeric C‐terminal structural domain; and the C‐terminal disordered tail region (CIDR), with the junction domain and the CIDR highly disordered [161]. It has been shown that the N‐protein of SARS‐CoV‐2 forms gel‐like biomolecular condensates and particles with viral RNAs that undergo phase separation, and phosphorylation promotes the formation of droplets comprising N‐protein viral RNA aggregates, while dephosphorylation facilitates granular aggregate formation [159]. Phosphorylation is important in regulating the state of N‐protein viral RNA aggregates, as unmodified proteins form structured oligomers suitable for nucleocapsid assembly, which protect the virus, while phosphorylated proteins form liquid‐like compartments important to viral genome processing, ensuring viral transcription in the host [159]. In addition, the formation of N‐protein and viral RNA bioconjugates is associated with host inflammatory responses induced by coronaviruses [160]. The formation of N‐protein viral RNA condensates in a host recruits TAK1 and IKK complexes, key kinases of NF‐κB signaling, to enhance NF‐εB activation. By inhibiting the formation of this condensate, 1,6‐HD inhibits coronavirus activation of host NF‐κB [160].

As previously mentioned, coronaviruses not only undergo phase separation, which enables separation of viral RNA, but also LLPS with some host proteins. Studies have shown that the N‐protein interacts with stress particle proteins [162] to inhibit SG formation. Additionally, the N‐protein can undergo phase separation to be sequestered from human granule‐associated heterogeneous nuclear ribonucleoproteins (hnRNPs), including TDP‐43, FUS, and hnRNPA2, in vitro. Many host cytoplasmic proteins, such as these hnRNPs, may act as scaffolds that facilitate the formation of multicomponent N‐RNA condensates to enable or accelerate viral replication [161]. In host cells, N‐protein LLPS that sequesters it from RNA inhibits the innate antiviral immune response by suppressing Lys63‐linked polyubiquitination and the aggregation of MAV. Disruption of N‐protein LLPS has been demonstrated to inhibit SARS‐CoV‐2 replication and rescue innate antiviral immunity [158].

In summary, phase separation is vital to infectious diseases and the cellular immune response. Disruption of viral‐specific condensate formation, protection of the normal function of cell intrinsic MLOs, and increased efficacy of membrane‐associated condensates that promote immune signaling may be approaches to treat infectious diseases.

### 5.5 Cancer

The etiology of cancer is extremely complex and includes viral infection, physicochemical factors, genetic factors, lifestyle, and other causes [163, 164]. Studies have demonstrated a close relationship between cancer and cellular phase separation. Phase separation is involved in oncogenic processes, including the regulation of cancer‐related signaling pathways, telomere aggregation in cancer cells, cancer‐related transcriptional dysregulation, and the proliferation of cancer‐related viruses in host cells. An increasing number of MLOs have been shown to be associated with oncogenesis; these include TP53 aggregates, SPOP‐DAXX bodies, SGs, and ALT‐associated promyelocytic leukemia (PML) nuclear bodies (APBs) [15]. Thus, phase separation provides new ideas to explain cancerogenesis and to develop cancer therapies.

#### 5.5.1 Regulation of Cancer‐Related Signaling Pathways

Cyclic adenosine monophosphate (cAMP) is an important second messenger in living organisms that broadly regulates cellular behavior and function. Research has shown that the type I regulatory subunit of cAMP‐dependent protein kinase (PKA), RIa, undergoes LLPS with cAMP to form biomolecular condensates, which are important for the maintenance of normal cell proliferation and cell transformation. Disruption to RIa LLPS using a PKA fusion oncoprotein associated with atypical liver cancer contributed to tumorigenesis [165]. The TCR signaling pathway in immune cells also undergoes phase separation and forms dynamic membrane‐associated clusters when TCR is activated [166]. As the TCR is similar to ligand‐activated receptor tyrosine kinases (RTKs), which are frequently mutated in cancer cells [167], it has been speculated that RTKs may, similar to TCRs, undergo LLPS and thus activate oncogenic downstream signaling events.

#### 5.5.2 Transcriptional Dysregulation

Phase separation is involved in transcriptional dysregulation in tumor cells. A characteristic feature of tumors is dysregulated transcription. MYC is a widespread transcription factor, and transcriptional dysregulation of MYC is frequently found in tumor cells, leading to tumorigenesis [168]. Relevant research has shown that LLPS is involved in the activation of the MYC superenhancer in host cells after Epstein–Barr virus (EBV) infection. The EBV proteins EBNA2 and EBNALP, which mediate virus and cellular gene transcription, are transcription factors that can form liquid‐like condensates at superenhancer sites in MYC and Runx3, causing the development of EB‐related tumors [169].

#### 5.5.3 Telomere Lengthening

Certain oncoproteins affect the lengthening of telomeres by affecting phase separation in normal MLOs, leading to cancer. Telomeres are repetitive sequences at the end of chromosomes. Telomeres in normal cells continue to shorten with mitosis, and their shortening is accompanied by cellular senescence and apoptosis [170]. However, some cancer cells, such as acute promyelocytic leukemia (APL) cells, maintain replicative potential by actively lengthening telomeres. APL cancer cells, which are telomerase‐free cancer cells, depend on APBs to lengthen telomeres. This mechanism is called the alternative lengthening of telomeres (ALT) pathway. APBs are pathological MLOs formed through LLPS when telomere DNA in APL cancer cells is damaged. APBs contain proteins that participate in homologous recombination, including replication protein A (RPA), Rad51, and breast cancer susceptibility protein 1 [171]. Moreover, new telomere DNA synthesis has been detected in APBs [172]. All this evidence suggests that APBs promote telomere synthesis. The formation of APBs drives telomere clustering [173], which leads to ALT pathway activation. Disruption to APB formation by knocking down PML protein expression leads to telomere shortening [15].

#### 5.5.4 Oncoprotein

In addition to oncoproteins that cause cancer development by affecting telomere length, some oncoproteins undergo LLPS and cause cancer by affecting intracellular other oncogenic factors. For example, in addition to APBs, the destruction of PML nuclear bodies (NBs) is evident in APL cells. Proteins in PML NBs are important to tumor suppression [174]. The oncoprotein PML/RARα leads to the disruption of PML NBs. Neddylation of the RARα moiety in PML/RARα induces aberrant phase separation in cancer cells and enhances RARα DNA‐binding ability, which hinders the normal phase separation of this PML moiety. The deneddylation of PML/RARα via MLN4924, a deneddylation drug, reverses this process and inhibits PML/RARα‐driven leukemogenesis [175].

#### 5.5.5 Cancer‐Associated Viruses

Cancer‐associated viruses invade host cells and induce cancer through phase separation [169]. The relationship between phase separation and viruses is described above, and the relationship between phase separation caused by cancer‐associated viruses and the oncogenesis of host cells cannot be ignored. To determine whether other cancer‐associated viruses, such as HPV and hepatitis B virus, induce cancer in the host via phase separation, future research is required.

## 6 Regulation of Phase Separation as a New Therapeutic Approach

### 6.1 PTM‐Related Drugs

Aberrant PTMs of many proteins often cause changes in phase separation rates; for example, TDP‐43 protein acetylation at lysine‐136 is likely to lead to insoluble aggregate formation, which recapitulate PTMs found in ALS patients. The deacetylating drug sirtuin‐1 can deacetylate K136‐acetylated TDP‐43 and reduce its aggregation propensity in cells [176].

### 6.2 Cellular Microenvironments of Drug Action

Studies have shown that certain protein condensates provide microenvironments for certain small‐molecule drugs to exert their effects. Small‐molecule antitumor drugs that target transcriptional regulators such as cisplatin and mitoxantrone and their targets, which have been proposed to be the estrogen receptor, CDK7, and BRD4 enriched in the nucleus and the extracellular MED1 protein, to form transcriptional condensates and exert drug activity. In vitro, deletion of the MED1 protein results in the loss of DNA cisplatinization. Furthermore, the mutation and overexpression of the MED1 protein are associated with tamoxifen resistance [18]. Therefore, drug resistance may be associated with abnormal phase separation of the proteins that form locations for drug effects.

### 6.3 Phase Separation of Nonproteinous Molecules

Most research on phase separation has been based on proteins. However, phase separation of certain small molecules is equally important. For example, lipid nanoparticle (LNP)‐packaged mRNA vaccines are the most extensively used vehicles for packaging, protecting, and delivering mRNA inside cells, and they are currently employed as delivery vectors for LNPs composed of multicomponent lipid systems, such as those with an ionizable lipid, a phospholipid, cholesterol, and a polyethylene glycol‐lipid compound. Studies have shown that different cholesterol analogues alter the LMP structure by changing the phase separation rate of their lipid components, improving mRNA transfection [177].

### 6.4 Cancer Treatment

We have revealed that oncogenesis closely links with phase separation including the regulation of cancer‐related signaling pathways, telomere aggregation in cancer cells, cancer‐related transcriptional dysregulation, and the proliferation of cancer‐related viruses in host cells. Focusing on the phase separation‐associated oncogenic processes, it may be possible to inhibit oncogenesis by interfering with LLPS. For example, the dysregulation of SHP2, a nonreceptor protein tyrosine phosphatase (PTP), is associated with several cancers [178, 179, 180]. When exposed to specific factors such as EGF and FGF, wild‐type SHP2 can form biomolecular condensates. Interestingly, oncogenesis‐associated SHP2 mutants have a higher propensity to form biomolecular condensates, promoting MAPK signaling and thus leading to ERK1/2 activation [181, 182]. SHP2 mutation‐induced LLPS can be suppressed by allosteric inhibitors of SHP2 including AB‐3068, TNO155, RMC‐4630, and RLY‐1971 [181]. These allosteric inhibitors are currently under evaluation in clinical trials for cancer treatment [179].

### 6.5 Drug Resistance

The emergence of drug resistance is closely associated with the formation of drug‐resistant biomolecular condensates in target cells after using drugs. Research has shown that inhibiting the formation of drug‐resistant biomolecule condensates through certain drugs can effectively suppress the development of drug resistance. For example, PD‐L1 therapy for treating tumors is prone to developing IFN‐γ‐mediated adaptive resistance. The emergence of drug resistance relates to the promotion of nuclear translocation and phase separation of YAP in tumor cells. The disruption of YAP phase separation reduces tumor growth, enhances the immune response, and makes tumor cells sensitive to anti‐PD‐1 therapy [183].

A similar situation also occurs in prostate cancer patients undergoing antiandrogen treatment. Androgen receptor (AR) can form transcriptionally active condensates through phase separation after being activated by androgens. Mutations in AR can lead to patients with castration‐resistant prostate cancer acquiring resistance to most drugs. Antiandrogens such as ET516 can disrupt AR condensates by inhibiting AR phase separation, effectively suppress AR transcriptional activity and inhibits the tumor growth of prostate cancer cells expressing AR‐resistant mutants [184].

### 6.6 Limit Inflammation

Phase separation in macrophages can regulate macrophage‐induced inflammation. By inhibiting the formation of phase‐separated TRAF6 droplet and preventing NF‐κB activation upon LPS stimulation, it can inhibit inflammation induced by macrophage activation. Sufu can suppress the formation of TRAF6 droplets and thus prevent the activation of NF‐κB upon LPS stimulation [185].

### 6.7 Antivirus

Phase separation can also be a new paradigm for antiviral drug research [186]. For example, cyclopamine can impede the replication of the human respiratory syncytial virus (RSV) by disrupting IB condensates. Moreover, the effectiveness of cyclopamine has been demonstrated in a mouse model, which reveals its therapeutic potential against the virus [187]. Similarly, drugs CVL218 and PJ34 can combine with SARS‐CoV‐2 N protein and diminish the local density of SARS‐CoV‐2 N‐nsp12 condensates, thus enhancing the permeability of the virus. With the help of CVL218 and PJ34, other small‐molecule drugs can enter the condensates more quickly. The combination of CVL218 with remdesivir can enhance the efficacy of remdesivir in treating SARS‐CoV‐2 [188].

## 7 Phase Separation in Other Organelles

In this article, we mainly introduced the phenomenon of phase separation that occurs in cell mitochondria. However, the phenomenon of phase separation is also observed in other organelles such as the endoplasmic reticulum, Golgi apparatus, and lysosomes (Table 2).

**TABLE 2:** Summary of phase separation in organelles except mitochondrion.

Organelle	Phase separation‐related molecules/structures	Role and mechanism of phase separation	References
Endoplasmic reticulum	AGO proteins, PI(4,5)P₂ lipids, IRE1α, FIP200	AGO interacts electrostatically with PI(4,5)P₂ to undergo phase separation, recruiting Ltn1 to catalyze ubiquitination of nascent peptides and collaborating with the VCP complex to degrade abnormal proteins. Under stress, ERES components form Sec bodies via phase separation, regulating protein secretion during stress and recovery. Phase separation of IRE1α into clusters is associated with SGs assembly, while FIP200 phase separation triggers ER‐mediated autophagy.	[189, 190, 191, 192]
Golgi apparatus	GM130, Golgins, GRASP, and other Golgi matrix proteins (GMPs)	GMPs form biomolecular condensates through phase separation, potentially involved in protein sorting and lipid metabolism in the Golgi, though specific mechanisms remain unclear.	[193, 194]
Lysosome	p62, HSP27	Abnormal FUS aggregates are recognized and cleared by lysosomes. Upon lysosomal damage, p62 forms condensates to recruit autophagosomes, a process regulated by HSP27 phosphorylation.	[195, 196]
Ribosome	Ribosomes themselves do not exhibit phase separation, but ribosomes regulate intracellular phase separation	The mTORC1 pathway modulates the effective diffusion coefficient of biomolecular condensates by regulating ribosome concentration, influencing intracellular phase separation in vitro and in vivo.	[197]

### 7.1 Phase Separation and Endoplasmic Reticulum

Endoplasmic reticulum (ER), the largest intracellular membrane system, serves as the organelle with the highest metabolic activity and is accountable for lipid synthesis. Research has observed the phase separation phenomenon in ER membranes of living cells and tissues by using in situ NIR ratiometric imaging [189]. The lipids on the endoplasmic reticulum can mediate the phase separation of argonaute (AGO) proteins, thus controlling nascent‐peptide ubiquitination. The electrostatic interactions between AGOs and the lipid PI(4,5)P2 mediated the phase separation on the ER. The AGO condensates located on the ER recruit the E3 ubiquitin ligase Ltn1, which catalyzes the ubiquitination of nascent peptides. Moreover, these AGO condensates coordinate with the VCP–Ufd1–Npl4 complex to handle unwanted protein products for proteasomal degradation [190]. The phenomenon of phase separation in the endoplasmic reticulum has also been observed during the formation of Sec bodies. When cellular stressors are expressed in Drosophila and mammalian secretory cells, components of ER exit sites (ERES) turns into Sec bodies through phase separation, regulating protein secretion during periods of stress and stress relief [191].

In addition, the activation of ER‐related transmembrane protein kinase/endoplasmic reticulum inositol requesting enzyme 1 (IRE1) in the endoplasmic reticulum can form large clusters/foci through phase separation. In mammalian cells, formation of IRE1α clusters is closely related to the assembly of SGs [192]. The outer surface of the ER membrane can also serve as autophagosome initiation sites. Autophagy stimulation triggers ER‐mediated autophagy by activating calcium transients above to induce phase separation of FIP200 [192].

### 7.2 Phase Separation and Golgi

The Golgi apparatus plays a crucial role in protein sorting and lipid metabolism. The Golgi Matrix Proteins (GMPs), mainly including Golgins and Golgi Reassembly and Stacking Proteins (GRASPs), are vital to its structural integrity and organization. Research shows that GMPs in various Eukaryotic lineages exhibit a significant tendency to form biomolecular condensates through phase separation [193]. Golgins are peripheral membrane proteins of the Golgi, and GM130 is the most abundant Golgin at the Golgi. Research has shown that GM130 can undergo phase separation to form biomolecular condensates [194]. At the same time, GMPs such as the Golgins and the GRASPs also strongly tend to form biomolecular condensates through phase separation. However, the function of the phase separation phenomenon in the Golgi apparatus has not yet been studied. Studies indicate that LLPS of Golgi relate to the functions of protein sorting and lipid metabolism in the Golgi apparatus [193].

### 7.3 Phase Separation and Lysosomes

Lysosomes are also closely related to the biomolecular condensates formed by intracellular phase separation. Studies have shown that aggregates of FUS, but not fluid condensates, result in the accumulation of functional lysosomes [195]. This implies that the condensates formed by phase separation may perform physiological functions. When abnormal phase separation lead to the aggregation of FUS, lysosomes can recognize and remove them.

Phase separation also participates in the clearance of damaged lysosomes. When lysosomes in HeLa cells and neurons are damaged, the selective autophagy adaptor SQSTM1/p62 is recruited to damaged lysosomes. Research observed that p62 forms condensates on damaged lysosomes, facilitating the formation of autophagosomes. The formation of p62 condensates are regulated by the small heat shock protein HSP27, which is phosphorylated in response to lysosomal injury [196].

### 7.4 Phase Separation and Ribosome

Though phase separation phenomenon has not been observed in ribosome, studies have shown that ribosome may act as factors that regulate intracellular macromolecular crowding, regulating intracellular phase separation. mTORC1 pathway can modulate the effective diffusion coefficient of biomolecular condensates of specific size by tuning ribosome concentration. This change in ribosome concentration affects phase separation both in vitro and in vivo [197].

## 8 Perspective and Discussion

Studies on phase separation are still in an emergent stage, and many mysteries and opportunities remain to be discovered. Phase separation is related to salt ion concentration. To exclude the influence of salt concentration changes in vitro [198], the optimum salt ion concentration is often determined before an experiment is performed. In vivo, the ion concentration inside and outside the cell may vary under different conditions. For example, in hyperparathyroidism, increased parathyroid hormone promotes bone resorption, and entry of bone calcium into the blood increases the concentration of calcium ions in the patient's blood [199]. Whether these changes in blood calcium influence the phase separation of normal cells in the patient is a topic worthy of study. Changes in calcium ion concentrations are also evident during bone remodeling. Osteoclasts secrete enzymes that dissolve the bone matrix, which leads to an increase in local calcium ion concentrations. Similar alterations in salt ion concentration in the body may occur in many diseases, such as renal failure, hyperthyroidism, and aldosteronism. The relationship between pathologically induced salt ion concentration abnormalities and abnormal phase separation is an interesting topic of further research.

This article summarizes the LLPS of mitochondrial‐associated proteins. The LLPS of TFAM and mt‐DNA regulates the self‐assembly of mt‐nucleoid. The mutation of TFAM disturbs the normal formation of mt‐nucleoid, which may obstruct the transcription of mt‐DNA. The LLPS of RNA‐processing proteins and mt‐RNA regulate the formation of MRGs. MRGs participate in the mitochondrial posttranscriptional pathway, which is vital to the expression of mitochondrial genes. The deletion of disordered domains in RNA‐processing proteins and mt‐RNA can obstruct the formation of MRGs, which can be observed in dysfunctional mitochondria. Nur77 undergoes LLPS with p62/sqstm1, which can sequester dysfunctional mitochondria and deliver targeted mitochondria bound for mitophagy. In bacteria, the LLPS of RNase E with mRNA forms BR bodies thus facilitating the rapid mRNA decay process, which is vital to the growth of bacteria. The phenomenon of mitochondrial LLPS is still in the exploratory stage, which demonstrates that the dynamics of LLPS is critical for mitochondrial function. The disturbance of mitochondrial associated protein LLPS can cause severe damage to mitochondrial function. The relationship between phase separation and mitochondria also deserves studying. As ATP inhibits the formation of biomolecular condensates, inhibition of mitochondrial ATP synthesis led by mitochondrial abnormalities should influence intracellular phase separation [53]. Mitochondria are mainly production sites of ATP. More and more evidence point out that mitochondria are in close communication with the cytoplasm and nucleus [36]. Here, we speculate that mitochondria and mitochondrial energy metabolism may closely link to the regulation of intracellular phase separation. However, relevant research is far from adequate.

As mentioned above, condensates formed on the cell membrane by certain proteins and lipids are vital for signal transduction mediated via signaling molecules in the membrane. Lipids on the membranes of membrane‐bound organelles in cells similarly mediate the phase separation of proteins. As mentioned previously, the lipids on the endoplasmic reticulum can mediate the phase separation of AGO proteins [190].

Research has found that lipid droplets in cells can serve as substrates for the phase separation of some proteins. In other words, the surface of lipid droplets, which are energy‐storage organelles in the cell, provides a site for IDPs to undergo phase separation and form molecular condensates. For example, the model disordered domains FUS LC and LAF‐1 RGG separate into protein‐rich and protein‐depleted phases on the surfaces of lipid droplets [200]. Whether there are more substances involved in phase separation, such as carbohydrates and ATP, is also a direction for future research.

Phase separation abnormalities are likely common, as point mutations in proteins alone can disrupt intracellular condensate formation [8, 52]. Therefore, many systemic diseases may be associated with phase separation abnormalities. However, recent research on phase separation and disease has been limited. Phase separation‐related diseases are often associated with protein mutations and PTMs [201]; therefore, researchers need to distinguish whether protein mutations or PTMs cause disease or whether phase separation abnormalities cause disease, as protein mutations and PTMs often influence the functional domains of a protein. In regard to the methods that affect the phase separation of proteins inside and outside a cell, the effect of 1,6‐HD on chromatin in living cells has been shown to be irreversible [202]; the use of 1,6‐HD also directly impairs kinases and phosphatases independent of LLPS [203]; and trans‐cyclohexane‐1,2‐diol and 1,6‐HD both show significant cytotoxic properties. Thus far, it is not possible to control the variables in LLPS experiments perfectly; therefore, exploring more moderate tools is a direction for future research.

Cells always face the choice of maintaining their current state or transforming into another state. The maintenance or change of such state is regulated by various intracellular and extracellular factors. The interaction of various factors endows the fate of cells with the characteristics of variability and transformation, which is called cell plasticity control. Microtubule cytoskeleton plays an important role in regulating cellular plasticity as it participates in cell migration, cellular shape, mitosis, intracellular transport, and cell polarity [204]. End‐binding (EB) protein, an important part of microtubule cytoskeleton, is vital for cellular dynamics and organelle movements [205]. Research shows that EB1 forms biomolecular condensates to track microtubule plus‐end. The disturbance of EB1 LLPS ability can affect microtubule growth and the frequency of catastrophe which impact cell plasticity control [206].

In summary, the study of phase separation phenomena is still full of challenges and opportunities. The current research on phase separation mainly focuses on proteins, but the discovery of lipid phase separation phenomenon indicates that other substances can also undergo phase separation. The expansion of phase‐separated substances is a research direction. Second, research on phase separation mainly focuses on in vitro experiments, lacking in vivo experiments. Under pathological conditions, patients often experience disturbances in water salt balance, which is a major factor affecting phase separation in vitro. How to design a series of systematic in vivo experiment for phase separation is also a major challenge. Finally, protein mutations or 1,6‐HD was often used to disturb the formation of biomolecular condensates. It is also difficult to distinguish whether the experimental results are caused by structural domain abnormalities of protein mutations, cytotoxicity of 1,6‐HD cells, or phase separation abnormalities. Finding better methods for phase separation is also a major challenge.

## Author Contributions

C.A.G., P.D., and J.J.G. conceived the review and drafted the initial manuscript. C.A.G. drew the figures and arranged the tables. J.J.G. and C.Q.Z. provided essential ideas to this work and revised the manuscript. All authors have read and approved the final manuscript

## Ethics Statement

The authors have nothing to report.

## Conflicts of Interest

The authors declare no conflicts of interest.

## Data Availability

The authors have nothing to report.
